# Applications of Ultrasound to Stimulate Therapeutic Revascularization

**DOI:** 10.3390/ijms20123081

**Published:** 2019-06-24

**Authors:** Catherine M. Gorick, John C. Chappell, Richard J. Price

**Affiliations:** 1 Department of Biomedical Engineering, University of Virginia, Charlottesville, VA 22908, USA; cmg6ae@virginia.edu; 2 Fralin Biomedical Research Institute at Virginia Tech Carilion School of Medicine, Roanoke, VA 24016, USA; jchappell@vtc.vt.edu

**Keywords:** arteriogenesis, therapeutic revascularization, ultrasound, microbubbles, biomaterials, drug and gene delivery

## Abstract

Many pathological conditions are characterized or caused by the presence of an insufficient or aberrant local vasculature. Thus, therapeutic approaches aimed at modulating the caliber and/or density of the vasculature by controlling angiogenesis and arteriogenesis have been under development for many years. As our understanding of the underlying cellular and molecular mechanisms of these vascular growth processes continues to grow, so too do the available targets for therapeutic intervention. Nonetheless, the tools needed to implement such therapies have often had inherent weaknesses (i.e., invasiveness, expense, poor targeting, and control) that preclude successful outcomes. Approximately 20 years ago, the potential for using ultrasound as a new tool for therapeutically manipulating angiogenesis and arteriogenesis began to emerge. Indeed, the ability of ultrasound, especially when used in combination with contrast agent microbubbles, to mechanically manipulate the microvasculature has opened several doors for exploration. In turn, multiple studies on the influence of ultrasound-mediated bioeffects on vascular growth and the use of ultrasound for the targeted stimulation of blood vessel growth via drug and gene delivery have been performed and published over the years. In this review article, we first discuss the basic principles of therapeutic ultrasound for stimulating angiogenesis and arteriogenesis. We then follow this with a comprehensive cataloging of studies that have used ultrasound for stimulating revascularization to date. Finally, we offer a brief perspective on the future of such approaches, in the context of both further research development and possible clinical translation.

## 1. Therapeutic Vascular Remodeling

The vascular system facilitates the transport of oxygen and essential nutrients to all tissues, aids in maintaining body temperature and tissue fluid levels, and removes metabolic waste byproducts, regulating each process through specific mechanisms for different physiological states. In the case of disease pathology, the vasculature can be driven from quiescence and can actively alter its structure, engaging in processes such as vasculogenesis (de novo vessel formation), angiogenesis (new vessels sprouting from existing vessels), arteriogenesis (collateral artery growth), and vessel regression (disassembly of vascular structures). Alzheimer’s disease, atherosclerosis (leading to peripheral arterial disease and myocardial ischemia), and osteoporosis are prominent examples of conditions characterized or influenced by insufficient vascularization or vessel regression [1]. Insight into the complex and highly integrated cellular, molecular, and genetic mechanisms underlying the aforementioned vascular growth and remodeling processes is steadily increasing through worldwide research efforts. In turn, there has been an expansion in the potential of clinically relevant therapies that seek to augment angiogenesis or arteriogenesis in states of vascular deficiency or regression.

Many current treatments for vascular disorders involve invasive surgical procedures that risk severe complications and are not available to all patients [2,3,4,5,6]. Therefore, with the goal of minimizing invasiveness and undesired side effects, novel cell-, molecule-, and gene-based interventions for therapeutic revascularization are being explored in a wide range of studies, all seeking the potential development of clinically relevant therapies. For instance, adult stem cells, i.e., adipose-derived cells, bone marrow-derived cells (BMDCs), and circulating endothelial progenitor cells (EPCs), have been investigated for their ability to promote enhanced vascular growth and remodeling in tissues afflicted by an ischemic injury [7,8,9,10]. Vascular growth factors and cytokines like vascular endothelial growth factor-A (VEGF-A) [11,12,13], platelet-derived growth factor-BB (PDGF-BB) [14,15], and granulocyte-macrophage colony-stimulating factor (GM-CSF) [16,17,18], and genes encoding various pro-angiogenic and pro-arteriogenic molecules [19,20,21], have also been explored in the context of tissue ischemia. Furthermore, our emerging understanding of the roles of various epigenetic factors (e.g., DNA methylation [22] and non-coding RNAs [23,24]) in regulating vascular growth and remodeling now offers opportunities for new therapeutic targets [25].

However, considerable limitations exist not only in designing revascularization therapies that influence only the intended molecular targets, but also in physically delivering the therapeutic agents only to the disease site in a way where unrelated tissues are not affected. For instance, adsorption from the gut to the bloodstream (i.e., oral administration) may pose risks to systemic tissues and organs, and intravenous injection and catheter-based administration methods face similar difficulties. Implanting controlled release devices can involve an invasive procedure and also present the risk of infection or an adverse biomaterial response. Direct injection of a therapeutic agent into and/or near ischemic tissue is perhaps the most commonly used mode of delivery for revascularization, but this invasive approach often lacks a sustained vascular remodeling response and can yield the poor dispersion of therapeutic agents away from injection site(s). Thus, in the context of therapeutic revascularization, substantial opportunities remain for developing more efficient delivery approaches that offer high spatial accuracy and minimal invasiveness.

## 2. Ultrasound Technology: Basic Principles and Contrast Agents

One minimally-invasive technology that has the potential to both achieve high spatial accuracy and yield wide therapeutic dispersion through tissue for revascularization is therapeutic ultrasound (US). Before discussing how ultrasound may be applied therapeutically for revascularization, we provide here a brief background on the basic principles that govern US and its potentially beneficial effects on tissue. An US waveform is transmitted into a region of interest when specific piezoelectric elements positioned on the face of a transducer are activated by an appropriate electrical signal. The generated acoustic energy propagates through the tissue, encountering regions of varying acoustic (i.e., mechanical) impedance. These mechanical heterogeneities modify the ultrasound beam through attenuation and diffraction as well as reflection and scattering. In diagnostic ultrasound imaging, reflected and scattered energy returns to the transducer, which now behaves as a receiver, converting the mechanical acoustic energy into electrical energy. A meaningful image can then be displayed when these electrical signals are processed appropriately.

After many years of use as a minimally invasive imaging modality, it was discovered that US in conjunction with gas-filled contrast agent microbubbles (which enhance blood echogenicity and, consequently, the contrast between tissues during an US exam) could also be used for other applications. These microbubbles (MBs) circulating within the bloodstream can be destroyed by US, facilitating the assessment of tissue perfusion by its correlation to MB replenishment in a given region [26,27]. Concerns about the possible deleterious bioeffects from ultrasonic MB destruction have fueled investigations into the impact of this phenomenon on surrounding tissues. Observations from these studies have shown that localized regions of microvessels experienced increases in permeabilization as indicated by red blood cell (RBC) extravasation from sites of intravascular US + MB interactions [28,29,30,31]. Based on these findings, significant interest was generated in possibly exploiting these US + MB-induced bioeffects for beneficial purposes including the direct stimulation of vascular remodeling through the bioeffects of ultrasonic MB activation as well as the targeted delivery of therapeutic agents.

## 3. Ultrasound Activation of Microbubbles to Facilitate Angiogenesis and Arteriogenesis

Interactions between relatively low-power US and circulating MBs elicit a range of bioeffects through mechanisms that remain only partially understood [33,34]. In addition to the increase in microvascular permeability discussed in the previous sections [29,30], hemolysis (i.e., RBC destruction) [35,36,37,38] and arterial vasospasms [39] have been shown to occur near sites of US + MB interactions. Additionally, cavitation of MBs by US may cause free radical production [40,41,42], heating [43,44,45], shockwave emanation resulting in microstreaming [46,47,48], and bubble fragmentation producing microjets [34,49]. These effects may individually or collaboratively impact the surrounding microenvironment to elicit the tissue level consequences (e.g., capillary disruptions, wound healing pathways, hemostasis, inflammation signaling pathways). Components of these pathways are known to be involved in modulating vascular remodeling; thus, it was postulated that selectively instigating these pathways, among others, through the targeted destruction of MBs with US might result in a localized neovascularization response in treated tissues.

**Figure 1:**
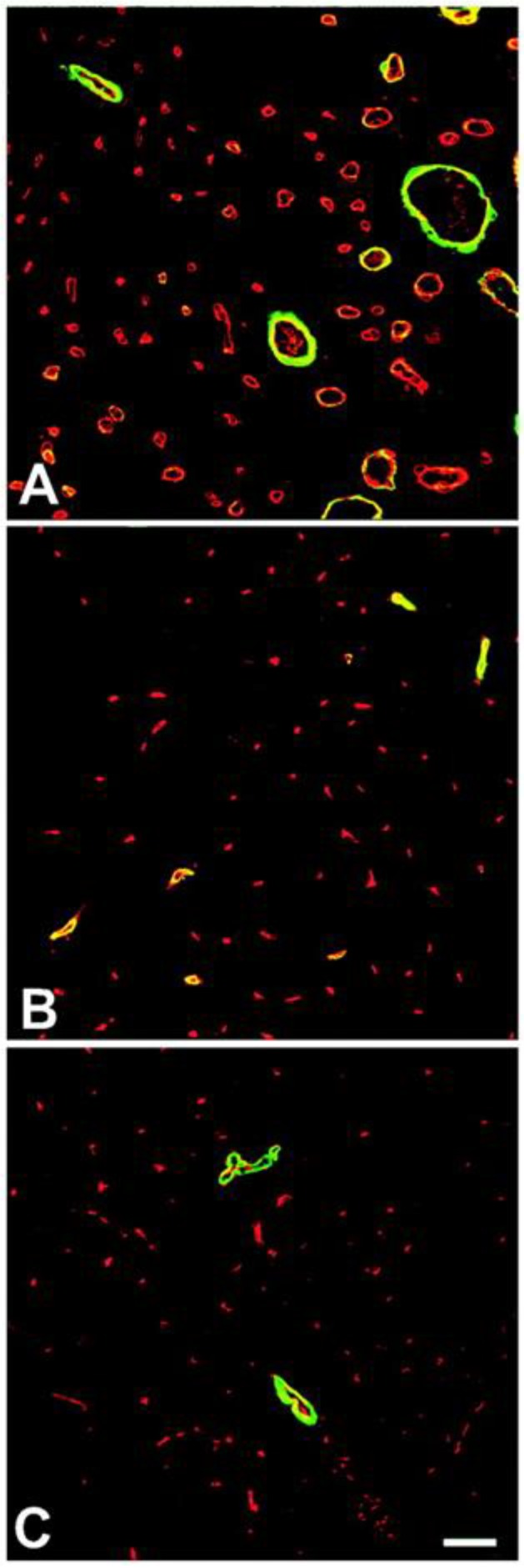
Activation of microbubbles with ultrasound can elicit arteriogenesis. Confocal micrographs illustrating the expression of SM α-actin (green fluorescence) in relation to microvessels, as labeled with BS-I lectin (red fluorescence). Images were taken 14 days after muscles were exposed to US-MB treatment (**A**) or sham treatment (**B**). Panel (**C**) represents the untreated muscle. Note the increased number and caliber of SM α-actin+ vessels with US + MB treatment (**A**). Bar = 30 μm. Adapted from Song et al. [32].

Indeed, this hypothesis was verified by an exploration of the vascular remodeling response in the gracilis muscle of a rat hindlimb exposed to low-power US and intravascular MBs [32]. Here, the application of 1-MHz pulsed US following intravenous injection of MBs induced capillary disruption sites as visualized by RBC extravasation. This elicited an arteriogenic response (Figure 1), which, in turn, enhanced blood flow in the hindlimb skeletal muscle. Furthermore, a follow-up study demonstrated the ability of this US + MB treatment scheme to significantly augment the vascular remodeling response of, and subsequently restore the perfusion to, a rat hindlimb affected by an arterial occlusion [50]. The experimental procedures from the rat studies were recapitulated in normal mice [51], in part to lay the foundation for studies addressing possible mechanisms behind the US + MB-induced neovascular adaptations. This transition to a mouse model provided a platform that was also advantageous for both mechanistic experiments involving genetic and/or cellular alterations and for experiments in a model of hindlimb ischemia. Although transient and failing to match the duration and extent of the response observed in the rat study, mouse hindlimb skeletal muscle exposed to a comparable US + MB treatment indeed exhibited a significant increase in neovascularization in comparison to the sham-treated muscles.

An investigation into the method by which these therapeutic vascular remodeling responses occur in the ischemic mouse and rat hindlimb revealed that the recruitment of CD18+ (integrin beta chain-2+) BMDCs is necessary for angiogenesis, arteriogenesis, and CD11b+ monocyte recruitment, as animals with CD18^-/-^ BMDCs did not exhibit vascular remodeling [52]. A separate study demonstrated that treatment with US + MBs induced the recruitment of CD45+ leukocytes including macrophages and T-lymphocytes to the treated tissue. Both of these cell types produce VEGF-A, and VEGF-A levels were elevated in the treated muscle, corresponding to increased capillary density, surface vascularity, blood flow, and functional improvement in the previously-ischemic skeletal muscle [53].

The selected parameters of the US + MB application have been shown to play a role in the ensuing vascular responses. For example, the influence of raising peak US rarefactional pressure to 3.8 MPa (in contrast to the previously described studies, which used pressures as high as 1.4 MPa), which ensures collapse of 100% of all MBs within the US focal region, has been tested. Animals treated at this high US pressure exhibited a marked decrease in capillary density immediately following treatment as well as clear evidence of hemorrhage. While capillary density increased somewhat in the weeks following treatment, it only reached 70% of baseline by 27 days post-treatment, suggesting that 100% MB collapse by high pressure US causes capillary destruction from which normal rats cannot recover [54]. Further study by this group explored the effects of different concentrations of MBs at a lower US pressure (0.7 MPa), and found that increased concentrations of MBs were associated with a greater degree of vascular permeability and VEGF expression [55].

While the majority of studies investigating the role of US activation of MBs to promote vascular remodeling have been in the context of skeletal muscle, several other tissues have also been investigated. US + MBs have been used to stimulate revascularization in the myocardium following acute myocardial infarction. In a mouse model, US activation of MBs resulted in increased microvascular density and reduced scar size, along with a transient up-regulation of VEGF-A and IGF-1 in the myocardium and improved left ventricular function [56]. Recently, there has been increased interest in the potential application of US and MBs to induce remodeling of brain vasculature. Focused US (FUS) in combination with MBs can be used to temporarily open the blood–brain barrier (BBB) at specific sites in the brain [57,58,59,60], consistent with the increased vascular permeability observed in other tissues. It has been observed that following disruption of the BBB by FUS and MBs, there is an acute upregulation of proinflammatory cytokine genes as well as angiogenesis-related genes in microvessels [61]. Further investigation of this phenomenon revealed that the transcriptional changes did in fact correspond with functional responses—following FUS activation of MBs in the hippocampus, there was a transient increase in blood vessel density as well as increased newborn endothelial cell density and frequency of small blood vessel segments [62].

## 4. Ultrasound and Microbubbles to Deliver Genes, Molecules, or Cells to Facilitate Angiogenesis and Arteriogenesis

**Figure 2:**
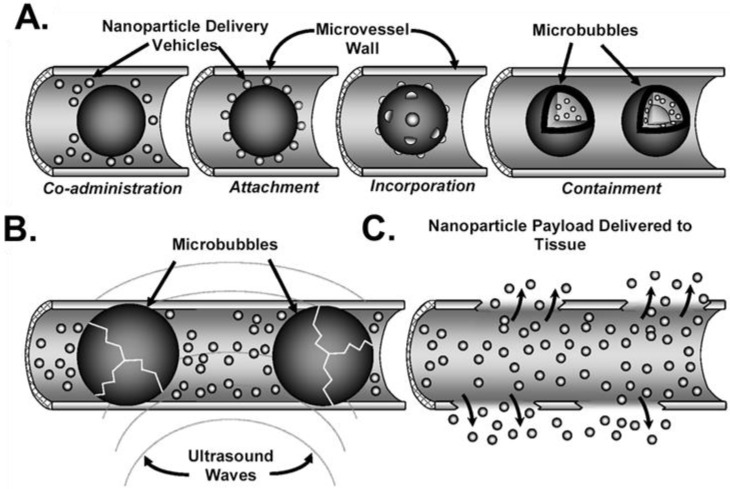
Overview of approaches to US + MB-mediated drug and/or gene delivery. (**A**) The therapeutic agent (nanoparticles in this example) may be co-administered with contrast agent microbubbles, attached to microbubble shells, incorporated into the microbubble shell, and/or contained within the microbubble. (**B**) The application of US activates the oscillation of microbubbles. At high enough peak-negative acoustic pressures, microbubbles can be fragmented. In some approaches, microbubble activation may lead to dissociation of a bound therapeutic from the microbubble. (**C**) Ultrasound–microbubble interactions simultaneously act to permeabilize the surrounding microvasculature, facilitating delivery of the therapeutic via diffusion and/or convection from the bloodstream to the ultrasound-targeted tissue. Adapted from Chappell and Price. [98].

As described previously, observations that US-mediated destruction of MBs could enhance blood vessel permeability have spurred interest in using these phenomena to facilitate the targeted delivery of therapeutic agents. The general premise is to co-administer a therapeutic agent, either freely circulating in the bloodstream alongside MBs, or physically associated with the MBs (i.e., bound/tethered to their surface or contained within the MB (Figure 2)) into the bloodstream while the MBs are circulating. Following US-induced cavitation of the MBs, there is increased permeability of the vessels at the site of US exposure, allowing for increased extravasation or uptake of the therapeutic agent in the circulation (Figure 2). Demonstrating this targeted delivery concept, Price et al. delivered polymer microspheres into the interstitium of rat spinotrapezius muscle via microvessel disruptions caused by ultrasonic MB destruction [31]. One of the first studies to show successful US + MB-targeted gene delivery involved the transfer of the P-galactosidase gene into rat myocardium through echocardiographic MB destruction [63]. While gene delivery to the myocardium with this US + MB technique has been investigated in several other studies [36,53,64,65,66,67,68], the potential treatment of various pathologies using US + MB-mediated gene transfer has also been explored in numerous other tissues including skeletal muscle [69,70,71,72,73,74], liver [75,76,77,78,79,80,81,82,83], and brain [84,85,86,87,88,89,90,91,92,93,94,95,96,97].

**Figure 3:**
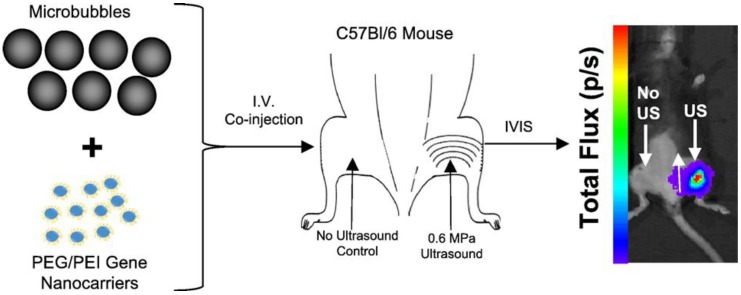
Overview of the strategy for eliciting ultrasound-targeted transfection of hindlimb adductor muscles via the delivery of non-viral gene nanocarriers. (**Left**) Contrast agent microbubbles are intravenously co-injected with gene-bearing nanocarriers. (**Middle**) Pulsed ultrasound is then applied to the adductor muscle group, which activates microbubble oscillation and facilitates nanocarrier delivery to the muscle tissue. (**Right**) Reporter gene expression is evident only where ultrasound has been applied. Adapted from Burke et al. [70].

Using US + MBs to facilitate nucleic acid delivery to specifically modulate vascular remodeling remains a relatively underexplored technology. The delivery of a VEGF-A gene to the rat myocardium has been demonstrated using US-targeted MB destruction. After treatment, increased levels of VEGF-A mRNA and protein were evident as well as increased capillary and arteriolar density within the myocardium [65]. The VEGF-A gene is also delivered to skeletal muscle in rats following a hindlimb ischemia surgery. In this study, the authors noted increased levels of the VEGF-A mRNA shortly after US + MB treatment as well as enhanced tissue perfusion, which was attributed to an observed increase in arteriolar density [73]. Moreover, it has been shown that genes may be delivered in a highly-targeted manner to hindlimb skeletal muscle using ultrasound in combination with non-viral gene nanocarriers (Figure 3) [70]. Although not yet utilized in a therapeutic revascularization capacity, this nanocarrier approach offers enticing options for technology development in this space going forward.

US and MB interactions have also been used to stimulate vascular remodeling in tissues other than skeletal muscle. One particularly interesting example entails the use of ultrasound to deliver a VEGF-A gene to the placental basal plate in a pregnant baboon to stimulate uterine artery remodeling [99]. Meanwhile, myocardium obviously also represents an important target for therapeutic revascularization. Delivery of a hepatocyte growth factor (HGF) plasmid to the myocardium in a canine model of myocardial infarction resulted in increased capillary density as well as reduced infarct size and scar tissue formation [100]. An HGF plasmid has also been delivered with US + MBs in a rat model of myocardial infarction. This treatment resulted in reduced left ventricular hypertrophy and scar formation as well as increased capillary and arterial density in the US-treated region [68]. Additionally, recent studies have explored the potential of the US + MB approach to deliver therapeutic genes to the brain. Following evidence that VEGF-A delivery to the brain promotes angiogenesis and functional recovery after ischemic stroke [101,102,103], US + MBs were employed to deliver a VEGF-A plasmid to the peri-ischemic region of the brain after infarction. The VEGF-A treatment reduced infarct size and apoptosis, and increased vessel density through stimulation of angiogenesis [92].

While not within the strict definition of “vascular remodeling” as applied in this review article (i.e., vasculogenesis, angiogenesis, and arteriogenesis), a few groups have investigated the use of US for targeted delivery in the context of neointimal hyperplasia, and such studies may offer insight into therapeutic revascularization strategies. In a handful of studies, US + MB-mediated approaches have been utilized to deliver an NFκB cis-element ‘decoy’ to inhibit neointimal hyperplasia development in arterial injury models. Delivery of this decoy in a rat carotid artery injury model was found to prevent the upregulation of intercellular adhesion molecule 1 (ICAM-1) and vascular cell adhesion molecule 1 (VCAM-1) in the neointimal area otherwise activated by arterial injury as well as the influx of macrophages and T-lymphocytes into the intima and media [104]. Similar observations were made following delivery of the ‘decoy’ in murine [105] or porcine [106] arterial injury models, suggesting the potential of this approach for minimizing neointimal hyperplasia following angioplasty or other coronary interventions. In addition, US + MB delivery of an siRNA against ICAM-1 has also been shown to suppress the development of neointimal formation following a murine arterial injury, and inhibit the accumulation of T cells within the injured artery [107].

The US + MB-targeted delivery method can be used to deliver more than just nucleic acids to stimulate therapeutic revascularization. Intramuscular injections of bone marrow mononuclear cells (BM-MNCs) have been demonstrated to promote angiogenesis and functional recovery of ischemic muscle in both animals [108] and humans [109], motivating the development of a noninvasive delivery system for these cells. In a rat model of hindlimb ischemia, intravenous injection of BM-MNCs immediately after US activation of MBs resulted in a significant enhancement in blood flow recovery, increased capillary and arteriolar density, and augmented collateral vessel formation [110]. US + MBs have also been used to deliver BM-MNCs to the myocardium in a hamster model of cardiomyopathy. US-mediated delivery of the BM-MNCs resulted in increased capillary density as well as expression of VEGF-A and FGF-2 by the myocardial tissue. The BM-MNCs were found to adhere to the US-targeted vascular endothelium within the myocardium, and endothelial progenitors within the BM-MNC population were shown to trans-differentiate into endothelial-like cells to repair US-stimulated endothelium and supply angiogenic factors (VEGF-A and FGF-2) to promote neovessel formation. The authors also observed reduced fibrosis and improved cardiac function and blood flow in the US + MB + BM-MNC treated group relative to the controls [111].

**Figure 4:**
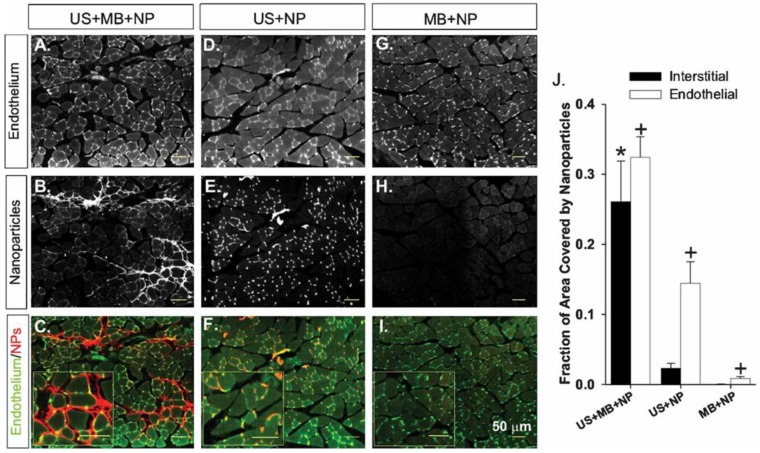
Muscle cross-sections illustrating nanoparticle (NP) delivery. (**A**–**I**) Representative images of sections taken from gracilis muscles treated with ultrasound (US) + microbubbles (MB) + nanoparticles (NP) (**A**–**C**), ultrasound + nanoparticles (**D**–**F**), and microbubbles + nanoparticles (**G**–**I**) are shown. Note the deposition of nanoparticles (red) in muscle treated with ultrasound + microbubble + nanoparticles. J: Bar graph representing the fraction of interstitial area (regions outside of muscle fibers and vascular structures) or endothelial cell area (cells comprising the walls of blood vessels) occupied by fluorescent polystyrene nanoparticles. Values are means with standard deviations. * Indicates significantly different (*p* < 0.05) to the interstitial area of all other groups. + indicates significantly different (*p* < 0.05) to the endothelial cell area of all other groups. Adapted from Chappell et al. [114].

**Figure 5:**
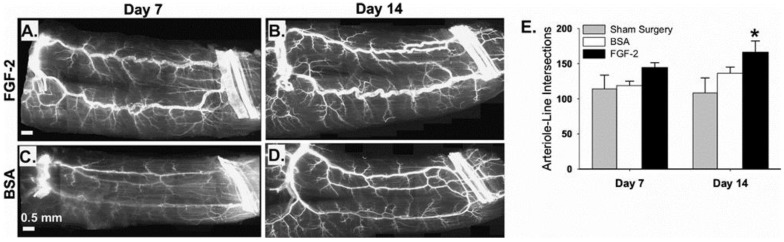
The delivery of FGF-2 bearing nanoparticles by ultrasonic microbubble destruction elicits arteriogenic remodeling in gracilis adductor muscle. (**A**–**D**) Representative whole-mount images of fluorescently-labeled SM α-actin+ vessels in gracilis adductor muscles seven and 14 days after FGF-2 (**A**,**B**) and bovine serum albumin (BSA) (**C**,**D**) treatment. Note the significant increase in arteriolar caliber and density in FGF-2-treated muscles. (**E**) Bar graph of arteriole-line intersections at both time points for FGF-2, BSA, and sham surgery treatment. Values are means with standard errors. * Indicates significantly different (*p* < 0.05) to BSA and sham surgery at day 14. Adapted from Chappell et al. [114].

US can also be used to deliver proteins. VEGF-A was delivered to the heart with US + MBs as early as 2000, although functional impacts on angiogenesis and arteriogenesis were not investigated [112]. In 2007, US + MBs were used in conjunction with granulocyte colony-stimulating factor (G-CSF) in the ischemic hindlimb muscles of mice. Here, instead of utilizing an intravenous injection strategy, G-CSF was injected subcutaneously. Specifically, mice were pre-treated with either a single or repeated subcutaneous injection of G-CSF after hindlimb ischemia surgery. One day following the final injection, the hindlimb muscle was targeted with US + MBs. Animals that received the G-CSF and US + MBs exhibited increased capillary density and collateral growth relative to animals that received only the G-CSF (no US) or just the US + MBs (no G-CSF) [113]. In 2008, we made use of an intravascular co-injection strategy, rather than pre-treatment. We first demonstrated the ability of US + MB interactions to facilitate the delivery of intra-arterially injected nanoparticles to the hindlimb skeletal muscle (Figure 4). Next, we injected nanoparticles containing FGF-2 intraarterially along with the MBs, and then used US to activate the MBs in the ischemic gracilis muscle of mice following hindlimb ischemia surgery. The therapy increased both the number and maximum intraluminal diameter of collateral arterioles (stimulating arteriogenesis, but not apparent angiogenesis) (Figure 5) [114].

While US provides a high degree of spatial targeting for treatment, modifications to the MB contrast agents themselves can provide an additional level of specificity. A number of studies have been conducted to modify the shell of the MBs to include targeting ligands, so that the MBs will selectively bind to particular regions of interest. For example, MBs coated with ligands that bind to P-selectin have been developed to target MBs (and thus, their imaging and therapeutic delivery effects) to the endothelium of inflamed blood vessels [115,116,117,118,119,120,121]. Other inflammation-related markers that have been used for targeting include E-selectin [122,123,124], ICAM-1 [125,126], and VCAM-1 [127,128,129,130]. A number of ligands against endothelial markers of angiogenesis including α_v_β_3_-integrins [131,132,133], VEGF receptor-2 (VEGFR2) [134,135,136,137], and endoglin [138,139] have also been used to target MBs to specific tissues or areas of the vasculature. While the majority of these studies have used the targeted MBs for diagnostic imaging purposes or the delivery of reporter genes or molecules, the method holds great potential for therapeutic applications related to vascular remodeling. Depending on the disease application, this approach allows for enhanced MB accumulation at the desired tissue site, permitting improved delivery of drugs and genes to promote angiogenesis or arteriogenesis.

## 5. Activation of Implanted Biomaterials with Ultrasound to Elicit Vascular Remodeling

One interesting new therapeutic application of ultrasound has been the advent of acoustically responsive biomaterials. For the past two decades, the principle of acoustic droplet vaporization (ADV) has been utilized to develop phase shift droplet emulsions, sub-micron-sized liquid droplets that vaporize into gas bubbles when exposed to sufficient acoustic pressure [140,141]. The general principle is to stabilize a perfluorocarbon with a relatively low natural boiling point (below body temperature, for example) in a superheated state using a surfactant, trapping the perfluorocarbon within the droplet, and preventing rapid aggregation. The speed of sound in the perfluorocarbon is substantially different than in the plasma surrounding the droplets in the bloodstream, permitting the use of the droplets as both contrast agents in an imaging setting as well as a therapeutic delivery mechanism [142]. Exposure to pressures above a particular threshold can induce the nucleation and growth of gas pockets within the droplets. The droplets then rapidly expand into gas bubbles considerably larger than their initial size [141,143,144,145], and can release payloads or oscillate like standard microbubbles. Emulsions of such droplets have since been engineered to encapsulate and deliver drugs in a targeted manner for a variety of disease applications [146,147,148,149,150].

Recently, these droplet emulsions have been utilized in a new application, droplet-hydrogel composite materials, where a hydrogel matrix is doped with a perfluorocarbon emulsion containing a therapeutic drug or molecule. This approach allows for both spatial and temporal control of the release of the therapeutic. In a 2013 in vitro study, a fibrin matrix containing perfluorocarbon droplets loaded with bFGF was activated with US, and the releasate from the hydrogel was applied to endothelial cells in culture. The bFGF-containing releasate did indeed stimulate metabolic activity in the cultured endothelial cells, demonstrating that the growth factor maintains functional bioactivity throughout the encapsulation and release processes. Increases in metabolic activity were also observed when endothelial cells were already seeded within the hydrogel at the time of US application [151]. These acoustically-responsive scaffolds (ARS) have also been shown to respond similarly to US in terms of payload release in vivo [152]. A bFGF-loaded ARS was injected subcutaneously into the dorsal region of mice, and later activated with US to release the growth factor. The mice that received the ARS and US demonstrated significantly enhanced perfusion relative to mice that received the ARS without US or a control fibrin hydrogel (with or without free bFGF). Additionally, the ARS + US group showed a significant upregulation in capillary density, indicating the growth factor-loaded ARS approach can be used to stimulate therapeutic angiogenesis [153].

## 6. Outlook

As reviewed here, over the past 15 years, numerous pre-clinical studies have demonstrated the potential of US to induce revascularization responses through the oscillation and/or destruction of gas-filled MBs, the delivery of therapeutic genes, proteins, or cells, or the activation of acoustically-responsive biomaterials. These approaches present opportunities for novel non-invasive and spatially-targeted treatments for diseases caused or characterized by insufficient or aberrant vasculature, replacing traditional interventions associated with invasive or high-risk procedures (surgery) or off-target effects (systemic delivery). In particular, we believe that US-mediated revascularization has immense potential for treating central nervous system disorders, where the blood–brain barrier poses a significant challenge to many treatment modalities as well as tissue engineering and biomaterials where the ability to non-invasively activate an implanted material would allow for highly tunable and versatile therapies that could be easily adapted to meet the needs of individual patients.

Nonetheless, it is also true that, despite this abundance of pre-clinical investigation, ultrasound-mediated approaches for revascularization have not been successfully translated to the clinic. While this is discouraging, the past few years have seen a handful of ultrasound-mediated drug delivery approaches enter into clinical trials, and we submit that these trials have the potential to open doors for many new applications going forward. This includes the use of ultrasound for stimulating revascularization. Indeed, in combination with i.v. microbubbles, ultrasound has now been used to safely open the blood–brain barrier in Alzheimer’s disease patients [154] and to deliver chemotherapy to patients with primary brain tumors [155,156]. Moreover, more trials utilizing focused ultrasound and microbubbles for the blood–brain barrier opening are just now getting underway, signaling an acceleration of clinical activity in this space. Meanwhile, ultrasound-microbubble-mediated drug delivery has also entered clinical trials for an application outside of the CNS, with the targeted delivery of gemcitabine having been performed in patients with pancreatic cancer [157]. With these trials serving as the foundation, we are hopeful that the knowledge gained regarding the safety and relative efficacy of ultrasound-microbubble-mediated treatments will facilitate the adoption of similar approaches for therapeutic revascularization. Overall, we argue that US-based methods of promoting angiogenesis and arteriogenesis still have potential to make an important positive impact on how we treat many vascular pathologies in the future.
